# Weight and Body Mass Index for Predicting Thyroxine Dose in Primary Hypothyroidism

**DOI:** 10.7759/cureus.15031

**Published:** 2021-05-14

**Authors:** Kashif Raashid, Osama Ishtiaq, Matiullah Kamin, Tejhmal Rehman, Sajjad Ali Khan, Umar Raja, Fazal H Shah

**Affiliations:** 1 Endocrinology and Diabetes, Shifa International Hospital, Islamabad, PAK; 2 Department of Medicine, Section of Diabetes and Endocrinology, Aga Khan University Hospital, Karachi, PAK; 3 Endocrinology, Shifa International Hospital, Islamabad, PAK; 4 Medicine, Tehsil Headquarters (THQ) Hospital, Bhakkar, PAK

**Keywords:** adult, body mass index, body weight, multivariate analysis, retrospective studies, thyroxine

## Abstract

Background

The treatment of primary hypothyroidism with thyroxine is weight-based or body mass index (BMI)-based. However, significant variation in the dose and the consequent delay in achieving euthyroid state is observed along the spectrum of patient body weights.

Objectives

To determine the weight and BMI-based dosing of thyroxine in primary hypothyroidism to achieve euthyroidism*.*

Material and methods

It was a retrospective review of the patient records conducted in the department of endocrinology, Shifa International Hospital, Islamabad, from July 1, 2014, to June 30, 2019 (five-year period)*. *Patients with clinical and biochemical hypothyroidism were enrolled and initiated on thyroxine replacement to achieve euthyroid status. A total of 504 patients were included in the study.

Results

The mean age was 44.5 ±13.6 standard deviation. Females were 83.5%. The mean dose of thyroxine to achieve euthyroid status was 107.7 ± 39.3 mean standard deviation mcg/day, i.e. 1.4 (0.5) mcg/kg/day. Euthyroid status was achieved in 264 (52.4%) of patients at three months. The mean TSH level after treatment was 2.09 (1.2) mU/L. The linear regression model showed that BMI and weight are independent predictors of the required thyroxine dose (R and Rsquare values are .274 and 0.075 for BMI and .319 and .102 for weight, respectively (P-value <.0001). There was no impact of age, gender, height, and duration of disease on achieving euthyroid at six months after treatment (P values: .85, .394, .827, and .105, respectively).

Conclusion

The optimum dose in primary hyperthyroidism can be determined with body weight and BMI-based calculations.

## Introduction

Globally, the standard treatment of primary hypothyroidism is through the replacement of thyroid hormone. Easily available and cheap levothyroxine (LT4) has virtually made every other option out of the arena for the treatment of primary hypothyroidism [1].

The other forms of thyroid hormone preparations, including desiccated extracts, are associated with variable and unpredictable bioavailability and, therefore, are inferior to oral levothyroxine in terms of efficacy, safety, and feasibility [2-4].

The correct dosing scheme of levothyroxine is pertinent since issues of over or under-dosage are associated with a variety of complications. Over replacement of thyroid hormone may lead to increased mortality, weight loss, decreased bone mineral density (BMD), and cardiac arrhythmias. On the other hand, inadequate dosing results in weight gain, declining metabolic rate, cardiovascular dysfunction, and lipid metabolism abnormalities [5].

Levothyroxine requirement in a hypothyroid patient is estimated by various parameters and is also dependent on the extent of baseline residual secretary function of the thyroid gland. Frequent monitoring of thyroid-stimulating hormone (TSH) levels can be used to assess the estimation of dosage efficacy but in practice, this is cumbersome due to increased laboratory cost and fluctuating negative feedback control of TSH. Consequently, attainment of euthyroid status may take up to two to three years in some patients. This can lead to an increase in health system costs due to frequent laboratory testing and physician visits. It is observed that an arbitrary dosing regimen, i.e. 100-150 mg/day, of levothyroxine achieves euthyroid status in nearly one-third of patients in the initial follow-up and frequently requires adjustment. The dosage of levothyroxine can be based on the weight of the patient and a current dosage of 1.6 to 2.1 mcg/kg/day may suffice for many patients [6]. In practice, the weight-based dosage is associated with frequent physician visits and TSH monitoring before the attainment of clinical and biochemical euthyroid status. BMI may provide a good estimate of levothyroxine dose, as it incorporates anthropometrics, which is easily applicable and measurable [7].

The levothyroxine dosage is affected by age, gender, menopausal status, thyroid pathology, and anthropometric measures. However, data regarding weight and BMI-based levothyroxine dosage regime for the South Asian population is limited. The data in our country regarding this specific aspect remains inconclusive [8].

The purpose of this study is to find and develop a simple algorithm to achieve euthyroid status by optimal levothyroxine dosage incorporating weight and BMI. The patient’s dissatisfaction is correlated with under or overtreatment [9].

The results of our study would help us to find a better predictor of the adequacy of levothyroxine dosage, which will lead to increase patient satisfaction and decrease healthcare burden.

## Materials and methods

We conducted a single Centre retrospective study in a large tertiary care hospital setting with the recruitment of patients using a hospital database. After the approval from the ethical review committee (IRB#298-788-2019), the data was extracted between July 1, 2014, up till June 30, 2019. A non-probability convenience sampling technique was used. The inclusion criteria were patients of either sex older than 18 years of age and BMI<50 kg/m^2^ with a diagnosis of primary hypothyroidism (TSH>10 mU/L). Following patients were excluded from the study: patients with a history of thyroid cancer, gastric bypass surgery, thyroid surgery, intake of liothyronine (T3) preparations, irregular follow-ups, changing brands of levothyroxine, and ongoing pregnancy. The study was approved by the local hospital ethical board. After fulfilling the inclusion and exclusion criteria, 504 patients were selected from the hospital database. Demographic details, history, clinical examination, and laboratory records were retrieved. Following the initiation of levothyroxine (1.6 mcg/kg/day), the TSH levels were measured at six to eight-week intervals until euthyroid status (TSH 0.4-4.5 mU/L) was achieved.

Statistical analysis

Quantitative data like age, weight, BMI, levothyroxine dose, time to achieve euthyroid status were expressed as mean and standard deviations. The qualitative data like gender and euthyroid achievement were expressed as frequency and proportions. To identify the impact of age, gender, BMI, educational status, and socioeconomic status on levothyroxine dosage, multivariate logistic regression was performed. The chi-square test and student t-test were used for binary comparison. Euthyroid dose and weight/BMI were analyzed via a quadratic equation. The best fit was used to determine the formula from the plotted curve. All the data were analyzed using Statistical Package for the Social Sciences (SPSS) version 25 for Windows (IBM Corp., Armonk, NY). The p-value <0.05 was considered statistically significant.

## Results

The data of 504 patients met the inclusion and exclusion criteria. The mean age was 44.45 ± 13.60 years. The mean weight, height, and BMI were 76.19 ± 15.38 kg, 160.93 ± 7.85 cm, and 29.44 ± 5.72 kg/m^2^, respectively. The mean duration of disease was 6.52 ± 5.36 years with a preponderance of female gender (83.5%, n=421). The dose of thyroxine to achieve euthyroid status was 107.70 ± 39.35 mcg/day (1.44 ± 0.528 mcg/kg/day). The euthyroid status was achieved in 264 (52.4%) of patients at three months and in all patients by the end of six months with a mean duration of treatment follow-up of 8.82 ± 3.365 months. The average TSH level after treatment was 2.09 ± 1.18 mU/L.

**Figure 1:**
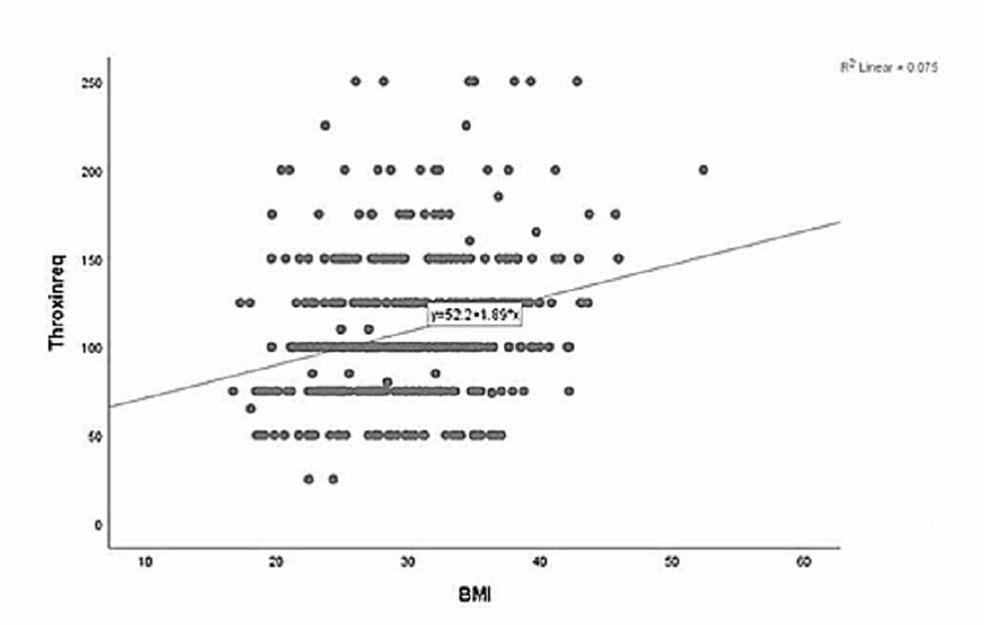
Linear regression plot BMI and thyroxine requirements BMI: body mass index

**Figure 2:**
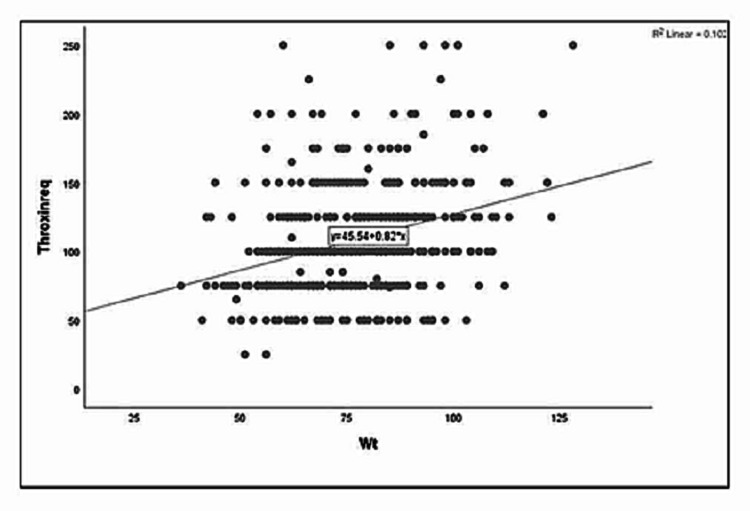
Linear regression plot weight and thyroxine requirements

The linear regression model showed that BMI is an independent predictor of the required thyroxine dose. The 0.274 is the correlation value with an R square of 0.075 (Figure 1). The R and R square values for weight are 0.319 and 0.102, respectively (Figure 2). The statistical significance is <0.0001 for both models of BMI and weight (Tables 1-2). The regression equations for the BMI, weight, and thyroxine dose is as follows (Figures 1-2). Thyroxine dose = 52.2 + 1.89 (BMI); thyroxine dose = 45.5 + 0.82 (weight).

**Table 1:** Euthyroid dose by BMI category BMI: body mass index, kg: kilogram, mcg: milligram

BMI (kg/m^2^)	Number of patients	Mean euthyroid dose (mcg/kg/day)	P-value
<22	38	1.797 ± 0.757	<0.0001^*^
22-28	184	1.511 ± 0.541	
28-32	125	1.403 ± 0.444	
>32	157	1.302 ± 0.451	
Total	504	1.441 ± 0.527	

**Table 2:** Euthyroid dose by weight category

Weight (kg)	Number of patients	Mean euthyroid dose (mcg/kg/day)	P-value
<50	17	1.787 ± 0.758	<0.0001†
50-70	171	1.609 ± 0.595	
70-90	227	1.340 ± 0.423	
>90	89	1.310 ± 0.471	
Total	504	1.441 ± 0.528	

Multiple logistic regression analysis was done and there was no impact of age, gender, height, and duration of disease on achieving euthyroid at 6 months after treatment (p values 0.85, 0.394, 0.827, and 0.105 respectively).

## Discussion

Hypothyroidism is caused by a deficiency of circulating thyroid hormones, and patients often present with a wide range of symptoms [10]. The main causes of hypothyroidism are autoimmune destruction, surgery, or radioiodine therapy. Some degree of hypothyroidism is present in the elderly population, of which most of the cases are subclinical [11]. Our study reveals that the mean age was 44.5 ±13.6 years and the mean dose of thyroxine to achieve euthyroid status was 107.7 ± 39.3, which was achieved at three months in a majority of patients. There was no impact of age, gender, height, and duration of disease on achieving euthyroid at six months after treatment.

The treatment is replacement with oral levothyroxine with an initial starting dose of 1.1 to 1.6 mcg/kg/day to achieve a euthyroid status, i.e. TSH levels of 0.4-4.0 mU/L [10]. The alternative preparations, such as injectable levothyroxine, are reserved for specific situations like malabsorption [12]. The dosage regimen of levothyroxine depends upon many factors, including body weight and BMI [1]. The demographic and dosing factors influence thyroid status [13]. Our study has predicted the correlation of levothyroxine dosage in primary hypothyroidism in the Pakistani population with patients’ anthropometric measures like body weight and BMI. A study by Mistry D et al. (2011) predicted levothyroxine dose correlation with body weight and BMI [14]. Their study also showed the impact of age ((P=.85) on levothyroxine dosage, which was not shown in our study Similarly, Ojomo KA et al. (2013) proposed a simple formula for levothyroxine dosage based on BMI, which was mcg/kg/day = -0.018*BMI + 2.13 [6]. In this study, the male gender was omitted due to inconsistency while in our study, the female gender was a dominant entity with 421 (83.5%). Multivariate analysis in our data showed that there was no effect of age on levothyroxine dosage requirement. The impact of age was seen in the study by Mistry D et al. (P<.01) but there was no impact of age in a study by Ojomo KA et al. [6,14].

Another study by Di Donna V et al. (2014) found a correlation of levothyroxine requirement and body weight, BMI, age, and preoperative mean corpuscular volume, which improved the euthyroid status up to 68% at the first visit within two months [15]. A meta-analysis by Zaborek NA et al. reviewing seven articles for proposing a dosing regimen for hypothyroidism showed that Poisson regression was significantly more accurate when based on weight and BMI. The correct prediction was 60.9% (P = .031). The traditional dosage regimen of 1.6 mcg/kg/day was accurate up to 51.3% [5]. Also, a recent study by Al-Dhahri SF et al. identified that body surface area was a significant predictor of levothyroxine dosage [16]. Their study also showed that the male gender required a higher dosage than females (p>0.05), which was not reproducible in our study (P = .394). Our study was limited due to data from a single endocrinology center and data were collected retrospectively.

Our study has a few strengths. First, this is one of the few studies specifically looking at a Pakistani population to predict weight and BMI based on the levothyroxine-dosing regimen. Second, we looked exclusively at patients with a diagnosis of primary hypothyroidism. Third, we tried to keep the levothyroxine preparations and formulations the same, as changing brands may lead to an unpredictable change in TSH as shown in another study [17]. The differences in optimal thyroxine dosage calculations based on various parameters and formulas have led to the adoption of a decision tree [18]. Euthyroid status is maintained throughout a patient’s life with continuous liaison between treating physicians. The changes in body weight, age, co-morbidities, and pregnancy require ongoing modifications in the dosage regimen. The signs and symptoms should be carefully assessed, as the overreliance on biochemical analysis of TSH is shifting now [8-9,19].

## Conclusions

Our study reveals that the optimal thyroxine dose to achieve the targeted euthyroid status in patients with hypothyroidism can be determined accurately with body weight and BMI-based calculations. Furthermore, our study reveals that there is no impact of age, gender, and duration of disease on achieving euthyroid status in primary hypothyroidism. Studies with a larger sample size would further validate the findings.
